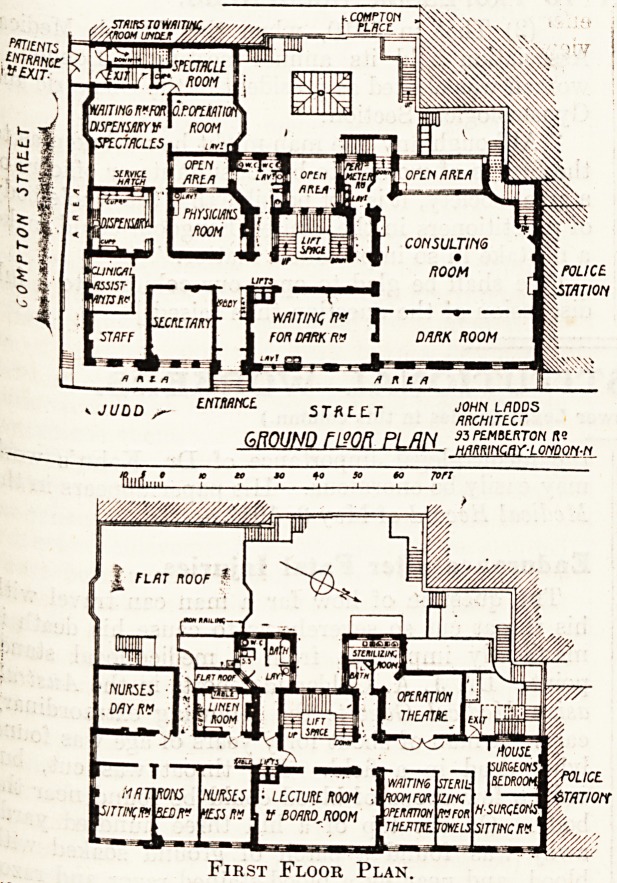# Central London Ophthalmic Hospital

**Published:** 1913-07-26

**Authors:** 


### HOSPITAL ARCHITECTURE AND CONSTRUCTION.

### Central London Ophthalmic Hospital.

This institution has recently been moved from Gray's Inn Road to a corner site in Judd Street, where a new building has been erected from the designs of Mr. John Ladds. The area of the new site is 10,000 square feet, and one side faces south-east to Compton Street, the main front in Judd Street facing north-east.

The building consists of five storeys, and on the basement floor nearly the whole area is covered over, only three small areas being left.

The out-patients' entrance and exit are both at the west end of the Crompton Street front. The entrance opens directly on to a staircase down to

First Floor Plan.

the basement, where there is a large top-lighted waiting hall; from this the patients ascend by another flight of stairs to the consulting room; leading out of this are two rooms—a waiting room for patients waiting to go into the dark room and the dark room itself. The method of dividing up this room is not shown on the plan; but from the size of the room we should imagine that some eight or ten patients could be seen simultaneously. From here the patients pass across the main staircase and hall to the medicine waiting room, or to the out-patient operating room or spectacle room, and so out to Compton Street.

The arrangement by which all the out-patients are brought past the main staircase and hall is an unfortunate one, and it would surely have been possible to have planned the building so that the out-patients were kept quite clear of the in-patient part of the hospital.

The pathological and bacteriological departments, with *x*-ray room, lecture room, museum, and students' work room are in the basement, where also are the kitchen offices. Inasmuch as there is only one staircase for all purposes, it is difficult to see how the kitchen offices can be effectively shut off from the laboratories and other rooms concerned solely with the scientific work of the hospital.

The wards at present in occupation provide accommodation for twenty-four beds—viz., two wards of ten beds each, two of two beds each. In the future provision will be made for forty beds.

Sisters and nurses are housed in separate rooms on the top floor.

## Figures and Tables

**Figure f1:**